# Integrated metabolomics and transcriptomics analysis reveals the potential mechanism by which Methyl jasmonate enhances the pungent flavor of soilless-cultivated Chinese chives (*Allium tuberosum*)

**DOI:** 10.1186/s12870-025-06410-3

**Published:** 2025-03-24

**Authors:** Cheng Wang, Jing Zhang, Jing Li, Qiang Chai, Jianming Xie

**Affiliations:** 1 State Key Laboratory of Aridland Crop Science /College of Agronomy, Gansu Agricultural University, Lanzhou, 730070 China; 2 College of Horticulture, Gansu Agricultural University, Lanzhou, 730070 China

**Keywords:** *Allium tuberosum*, Methyl jasmonate, Soilless culture, Cysteine sulfides, Flavor formation

## Abstract

**Background:**

Methyl jasmonate (MeJA) is an effective plant elicitor that enhances secondary metabolism. Chinese chives are prized for their pungent flavor, yet the biosynthetic pathways and regulatory mechanisms of flavor compounds induced by MeJA remain unclear.

**Methodology:**

This study integrated metabolomic and transcriptomic analyses to elucidate how MeJA modulates the biosynthesis of flavor substance precursors in soilless-cultivated Chinese chives.

**Results:**

MeJA treatment improved the dry matter content and nutritional quality of Chinese chives. We identified 36 volatile and 183 nonvolatile differentially abundant metabolites between the MeJA-treated and control groups. Gene expression analysis revealed 193 candidate genes associated with flavor formation. Among all the genes, a total of 2,667 DEGs were enriched primarily in metabolic pathways, including secondary metabolite biosynthesis, linoleic acid metabolism, and phenylpropanoid biosynthesis. Furthermore, exogenous MeJA inhibited the synthesis of endogenous jasmonic acid as well as enzyme activity and gene expression related to metabolic pathways. It also promoted the conversion of S-alkyl-L-cysteine to S-alk(en)ylcysteine sulfoxides (CSOs), increasing the accumulation of the flavor precursor CSOs and increasing the levels of S-methyl-L-cysteine. This led to increased concentrations of the key garlic flavor compounds methiin and alliin, intensifying the pungent flavor of Chinese chives. Notably, MeJA-induced *AtuFMO1* was associated with enhanced pungent flavor and may be regulated by AtuPHL7 and AP2/ERF-ERF transcription factors.

**Conclusion:**

In conclusion, exogenous MeJA activates key enzyme-encoding genes involved in the biosynthesis of garlic flavor precursor CSOs, leading to increased accumulation of the spicy compounds Methiin and Alliin. These findings establish *AtuFMO1* as a central hub linking hormonal signaling to flavor biosynthesis and provide molecular targets for improving Allium crop flavor and quality through precision horticulture.

**Supplementary Information:**

The online version contains supplementary material available at 10.1186/s12870-025-06410-3.

## Introduction

The genus Allium includes some of the oldest cultivated vegetables, which are prized for their ornamental qualities and use as spices and herbs [1]. Thiosulfates and reactive S-alk(en)yl cysteine sulfoxides (CSOs) give Chinese chives (*Allium tuberosum*) their characteristic aroma [2]. CSO biosynthesis in Allium sativum starts with sulfate assimilation, which involves high-affinity sulfate transport proteins (SULTRs) that transport sulfite. This product is converted to sulfide by ATP-sulfatases, adenosine-5’-phosphate sulfate reductase (APR), and sulfite reductase (SiR), leading to cysteine biosynthesis via O-acetylserine sulfhydrylase (OASTL) and glutathione formation through glutamate-cysteine ligase (GCL) and glutathione synthase (GS) [3]. Glutathione, a crucial organic sulfur compound in plants, initiates CSO biosynthesis through γ-glutamyl transpeptidase (GGT) and flavin-containing monooxygenase (FMO), which are involved in alliin synthesis [4]. Onions primarily contain isoalliin as their major CSO, while garlic contains alliin as its major CSO, with methiin serving as a secondary CSO in both species [5]. Despite the culinary prevalence of garlic and onion, research on the nutritional and flavor properties of Chinese chives, especially with respect to the biosynthesis and regulation of flavor compounds, is limited. Furthermore, in garlic, only γ-glutamyl transpeptidase and *S*-oxygenase involved in alliin biosynthesis have been identified [6]. Therefore, it remains challenging to generate plants with improved CSO levels or microbial systems for CSO synthesis through transgenic or gene-editing technologies [7].

Traditional soil-cultivated Chinese chives from *Bradysia odoriphaga* Yang et Zhang are vulnerable to safety and quality issues. Hydroponics offers a safer production method for Chinese chives, but the milder flavor of hydroponically grown chives poses a market challenge. The reduced pungency of hydroponic Chinese chives results from insufficient CSO accumulation, and FMO catalyzes sulfoxidation, the rate-limiting step in CSO synthesis [4]. Liu et al. reported that salt stress activates the transcription of the key enzyme-encoding gene *AtuFMO1*, increasing CSO accumulation [2, 8]. Yoshimoto et al. (2022) reported that CSO accumulation in Chinese chive callus tissue and the role of alliinase in CSO degradation [9]. CSO biosynthesis involves metabolic pathways involving valine, serine, cysteine, and glutamic acid [9]. The genes related to CSO synthesis identified in some Allium vegetables include plant cysteine synthase (PCS), GGT, and FMO [4]. However, research on their roles and regulation in Chinese chives is limited. Investigating the molecular mechanisms of CSO biosynthesis could improve the nutritional, flavor, and medicinal qualities of Allium vegetables, including Chinese chives.

Jasmonic acid (JA) signaling is crucial for regulating numerous physiological processes, including the production of defense metabolites and protein hydrolytic enzymes and the release of volatile organic compounds [10]. α-Linolenic acid is sequentially converted to 12-oxo-phytodienoic acid (OPDA) by lipoxygenase (LOX), oxyalkylene synthase (AOS), and oxyalkylene cyclase (AOC) in the plastid. Subsequently, 12-oxo-phytodienoic acid reductase (OPR) in the peroxisome transforms it into JA, which is further converted into methyl jasmonate (MeJA) by jasmonate o-methyltransferase (JMT) [11]. Active jasmonyl-isoleucine (JA-Ile) is recognized by the receptor COI1, triggering the degradation of the JAZ repressor and releasing the MYC2 transcription factor, activating the JA signal [12]. MeJA can consistently induce secondary metabolite production across the plant kingdom [13] and has been approved as a safe compound for use in all food products prior to harvest [14]. The application of MeJA considerably affects volatile compound biochemical pathways, enhancing the aroma of certain fruits and vegetables [15, 16]. Wang et al. reported that MeJA application increased the levels of sulfur-containing volatile compounds in hydroponically grown Chinese chives, increasing their pungency [17]. However, the metabolic regulatory network and mechanisms underlying the effects of exogenous MeJA on flavor quality and the biosynthesis of flavor precursor compounds in Chinese chives remain unclear.

Chinese chive is a tetraploid species with a 15 G per 1 C nuclear genome, slightly smaller than onion but 30 and 100 times larger than rice and *Arabidopsis thaliana*, respectively [18]. Compared to other vegetables like solanaceous crops, its genomic resources, including molecular markers and functional genes, remain limited [18]. Currently, many non-model plants, including Chinese chive, lack complete genomic data, preventing the establishment of reliable reference genomes [19]. Reference transcriptomes can be constructed through de novo assembly of RNA sequencing (RNA-Seq) data [19]. RNA-Seq, a promising application of next-generation sequencing (NGS) [20], is a cost-effective and widely utilized strategy [21] that has been successfully applied to analyze entire transcriptomes for genome-wide quantification of transcripts, identification of differentially expressed genes, development of molecular markers, and transcript annotation [22–24].

Analysis of transcript and metabolite profiles from elicitor-treated plants is effective for elucidating gene functions in secondary metabolite biosynthesis [13]. The molecular biology of special-flavor vegetables, including Chinese chives, has been largely neglected, resulting in limited data on the mechanisms underlying flavor quality. The identified volatile secondary metabolites contribute to flavor quality; however, the lack of transcriptional data restricts the use of biotechnological tools for crop improvement and understanding of flavor metabolic pathways. We used a multiomics approach integrating plant metabolomics with transcriptomics to understand the MeJA-mediated biosynthesis of flavor compounds, particularly gene expression changes, in Chinese chives. This study investigated the effects of exogenous MeJA on flavor formation in Chinese chives, with a focus on the key metabolic pathways involved in flavor compound biosynthesis. The impact of MeJA on metabolite accumulation and the expression of flavor-related genes in hydroponically cultivated Chinese chives was examined to elucidate the regulatory mechanisms of MeJA-induced CSO biosynthesis, providing insights into flavor formation and enhancement in hydroponically grown Chinese chives.

## Materials and methods

### Chinese chive plants and MeJA priming

The Chinese chive variety “Chive God F1,” cultivated by the Seed and Seedling Research Institute of Fugou County, Henan Province, was used as the experimental material. This variety is characterized by its strong disease resistance, cold tolerance. Seedlings were raised in the core demonstration area for Chinese chives in Wushan, China (N 34°250´-34°570´, E 104°340´-105°080´). One-year-old Chinese chives seedlings were transplanted into the substrate cultivation and hydroponic system in a glass greenhouse (20 ± 3 °C/15 ± 3 °C (day/night); relative humidity, 60-70%) at Gansu Agricultural University, Lanzhou, Gansu Province, P. R. China. The hydroponic system was prepared via an 11-well rectangular hydroponic tank, with a nutrient mixture prepared according to the methodology described by Wu et al. (2008) [25]. The substrate cultivation process involved mixing the cultivation substrate, perlite, and vermiculite in a 3:1:1 volume ratio within a 4 L plastic pot. A total of 352 plants were transferred to the hydroponic and substrate cultivation systems. A randomized design with three replicates was used in this study. The transplantation methods and nutrient management techniques employed in the substrate culture and hydroponic systems have been previously described [26].

At 21 days, Chinese chive seedlings, which had grown to 15–20 cm, were treated with MeJA (500 µM) via foliar spraying and applied evenly to the leaves every morning between 7:00 and 8:00 for seven consecutive days. The concentrations of MeJA were based on the concentration adopted by Wang et al. (2022) [27]. The S-MeJA (substrate cultivation) and H-MeJA (hydroponic cultivation) Chinese chives were treated with 150 mL of the specified MeJA solution daily, whereas the control groups (S-CK: substrate cultivation and H-CK: hydroponic cultivation) were sprayed with 150 mL of ultrapure water. The Chinese chives exhibited complete growth and reached commercial standards within a total growth cycle of 35 days. All treated chives were harvested, immediately frozen in liquid nitrogen, and stored at -80 °C for subsequent analysis. Each sample was obtained from at least 50 plants, and the resulting data were averaged from three independent biological replicates.

### Growth and nutritional quality analysis

The harvested samples were dried at 105 °C for 30 min, followed by 75 °C until constant weight, and dry matter content was recorded. Nitrate content was determined according to Cataldo et al. (1975) [28]. Vitamin C, soluble protein, and soluble sugar contents were measured using 2,6-dichlorophenolindophenol dye, Coomassie Brilliant Blue, and anthrone colorimetry [29–31], respectively. Total phenolics and flavonoids were quantified using Folin-Ciocalteu and sodium nitrite-aluminum nitrate assays [32, 33].

### Ultra-performance liquid chromatography tandem mass spectrometry (UPLC‒MS/MS) analysis of nonvolatile components

Chinese chive leaves were freeze-dried via a vacuum freeze-dryer (SCHENTZ-100 F) and ground in a mixer mill (MM 400, Retsch) at 30 Hz for 1.5 min. A 100 mg sample of the powder was dissolved in 1.2 mL of 70% methanol, agitated every 30 min for three hours, and stored at 4 °C overnight. The extract was subsequently filtered by centrifugation at 12,298 × g for 10 min. Chinese chive leaf extracts were subjected to analysis via an ultra-performance liquid chromatography (UPLC) system (SHIMADZU NexeraX2), which employs a SHIMADZU NexeraX2 apparatus. Metabolomic analysis, including the identification and quantification of metabolites [34], was performed by Wuhan MetWare Biotechnology Co., Ltd. (Wuhan, China) according to standard protocols. The analytical conditions included the use of an Agilent SB-C18 column (1.8 μm, 2.1 mm × 100 mm) and a mobile phase comprising 0.1% formic acid in water (solvent A) and acetonitrile (solvent B). The gradient commenced at 95% A and 5% B, transitioned to 5% A and 95% B over 9 min, and subsequently returned to 95% A and 5% B for a duration of 2.9 min. The flow rate was set to 0.35 mL/min, the column temperature was maintained at 40 °C, and the injection volume was 4 µL. The effluent was then connected to an electrospray ionization (ESI) triple-quadrupole linear ion trap (QTRAP) mass spectrometry (MS) system. Mass spectrometry was performed on an AB4500 QTRAP UPLC/MS/MS system (AB Sciex) equipped with an ESI Turbo ion spray interface, controlled by Analyst 1.6.3 software in positive and negative ion modes. ESI parameters included a turbo spray ion source, 550 °C source temperature, and ion spray voltage of 5500 V (positive)/-4500 V (negative). Gas settings were 50, 60, and 25.0 psi for GSI, GSII, and CUR, respectively, with high collision energy. The instrument was calibrated in QQQ and LIT modes using 10 and 100 µmol/L polypropylene glycol solutions. QQQ scans in MRM mode used nitrogen as the collision gas, with optimized DP and CE values for specific metabolites during each elution period.

### Headspace solid-phase microextraction gas chromatography–mass spectrometry (HS-SPME-GC‒MS) analysis of volatile components

In accordance with the methods of Wang et al. (2023) [17], HS-SPME-GC‒MS was used to detect volatile components in the substrate-cultivated and hydroponic Chinese chive leaves.

### Metabolic data analysis methods

The LC-MS platform, using Analyst 1.6.3 software, processed the mass spectrometry data, where the peak area (Area) of each chromatographic peak represented the relative abundance of the corresponding compound. The GC-MS platform processed the raw mass spectrometry data using Qualitative Analysis Workflows B.08.00 software for qualitative analysis, where the peak area of each metabolite was normalized to that of the internal standard ([2H3]-beta-Ionone). Metabolomic data were analyzed using R (http://www.r-project.org) to perform principal component analysis (PCA), hierarchical clustering analysis (HCA), Pearson’s correlation coefficient (PCC), and orthogonal partial least squares discriminant analysis (OPLS-DA) on data from all samples. The metabolite signal intensities were normalized and represented via color spectra. Before OPLS-DA, the data underwent log transformation (Log_2_) and mean centering. The variable importance in projection (VIP) values were extracted from OPLS-DA result. Metabolites with log_2_FC (fold change) ≥ 1, and VIP ≥ 1 were considered significantly regulated between groups. The identified metabolites were annotated via the Kyoto Encyclopedia of Genes and Genomes (KEGG, http://www.kegg.jp/kegg/compound/) and subsequently mapped to KEGG metabolic pathways (http://www.kegg.jp/kegg/pathway.html). The analysis of significantly regulated metabolites was conducted via metabolite pooling enrichment analysis (MSEA), with the level of significance determined through the application of hypergeometric tests.

### RNA extraction, detection, library construction, and sequencing

Total RNA was extracted from the samples using the TaKaRa Mini BEST Plant RNA Extraction Kit (TaKaRa, Beijing, China) according to the manufacturer’s instructions. The degradation and contamination of RNA were monitored via 1% agarose gel electrophoresis. The RNA purity was evaluated via a NanoPhotometer^®^ spectrophotometer (IMPLEN, CA, USA), and the RNA concentration was determined via a Qubit^®^ RNA Assay Kit and a Qubit^®^ 2.0 Fluorometer (Life Technologies, CA, USA). RNA integrity was evaluated via an RNA Nano 6000 Assay Kit on a Bioanalyzer 2100 system (Agilent Technologies).

A total of 1 µg of RNA was used to construct libraries with the NEBNext^®^ UltraTM RNA Library Prep Kit (Illumina). Approximately 200 base pairs of cDNA were subjected to screening with AMPure XP beads, PCR amplification, and purification to obtain the final library. The libraries were pooled in accordance with the requisite effective concentration and sequencing volume specifications for Illumina sequencing, yielding 150 bp paired-end reads. The raw data were subjected to filtration via Fastp v0.19.3 to remove reads identified as containing adapters. All subsequent analyses were based on the resulting set of clean reads. Clean reads were assembled via Trinity v2.11.0, and the transcripts were clustered and nonredundant via Corset (https://github.com/trinityrnaseq/trinityrnaseq). Gene expression levels were calculated using Fragments Per Kilobase of transcript per Million fragments mapped (FPKM).

### Transcriptome analysis

Differential expression analysis was performed using DESeq2 [35, 36] with unnormalized raw reads count data to identify differentially expressed genes (DEGs) in each sample. The Benjamini-Hochberg method was applied to correct the hypothesis testing probabilities (p-values) for multiple hypothesis testing, resulting in a False Discovery Rate (FDR). The criteria for selecting differentially expressed genes were|log_2_Fold Change| ≥ 1 and FDR < 0.05. The NR (Non-Redundant Protein Sequence Database, NCBI), NT (Non-Redundant Nucleotide Database, NCBI), Pfam (Protein Family Database), KOG/COG (Clusters of Orthologous Groups of Proteins), Swiss-Prot (UniProtKB/Swiss-Prot: manually annotated and curated protein sequences), and GO (Gene Ontology Database) were used for gene functional annotation and pathway analysis.

### Real-time quantitative PCR analysis

Total RNA was extracted from leaves via an RNA extraction kit (Acycrate Biotechnology Co., Ltd., China). A 2 µL sample was used for cDNA synthesis with the Evo M-MLV reverse transcription kit. Quantitative RT‒PCR was conducted via the SYBR Green Kit on a LightCycler^®^ 480 II with the following amplification conditions: 95 °C for 15 min, 95 °C for 10 s, and 60 °C for 30 s over 40 cycles. Each sample was tested in triplicate, and the relative gene expression levels were calculated via the 2^−∆∆CT^ method. The analysis was conducted via the statistical software package SPSS 20.0, and bar charts were generated via Origin Pro (2021). The housekeeping gene DN253_c0_g1 was used as the internal reference standard (Supplemental Table 23).

### Statistical analyses

Data analysis was conducted using SPSS 21.0 (IBM, Armonk, NY, USA). Significant differences (*p* < 0.05) were determined via ANOVA followed by Duncan’s multiple range test. Values are presented as the mean ± standard error of three biological replicates. Linear regression analysis was performed using Origin Pro 2021 (*p* < 0.05, *p* < 0.01). Figures were created using Origin Pro 2021 and Adobe Illustrator 2020. Gene expression was log_2_-transformed using FPKM values; when FPKM values were zero, FPKM + 1 was used for log_2_ transformation. The relative abundance of metabolites was represented as log_2_ values of chromatographic peak areas. Metabolite level and gene expression level FPKM values were Z-score normalized using the MetWare cloud platform (https://cloud.metware.cn) to generate heatmaps. Metabolite-level circos heatmap were processed and visualized via the Chiplot cloud platform (https://www.chiplot.online/), employing complete linkage for method selection, correlation for distance calculation, and Z score normalization.

## Results

### Growth and nutritional evaluation

The growth and nutritional quality of MeJA-treated Chinese chives in substrate and hydroponic media were assessed (Table 1). Exogenous MeJA application significantly increased dry matter, leaf vitamin C, and flavonoid contents while reducing leaf nitrate levels in soilless-grown Chinese chives. In addition, MeJA improved the soluble sugar and total phenol contents in the leaves of both hydroponically and substrate-grown Chinese chives.

**Table 1:** Effects of MeJA treatment on the growth and nutritional quality of hydroponic- and substrate-grown Chinese chives

Growth and nutritional indicators	H-CK	H-MeJA	S-CK	S-MeJA
Total dry matter (mg g^− 1^ FW)	104.5 ± 5.69c	118.19 ± 4.59b	115.27 ± 6.86b	128.56 ± 2.15a
Vitamin C (mg g^− 1^ FW)	0.28 ± 0.01c	0.39 ± 0.06b	0.39 ± 0.02b	0.41 ± 0.02a
Nitrate (mg kg^− 1^ FW)	479.71 ± 6.11a	390.24 ± 5.02c	467.65 ± 11.79a	429.17 ± 3.64b
Soluble Protein (mg g^− 1^ FW)	2.27 ± 0.01ab	2.34 ± 0.01a	2.21 ± 0.01b	2.13 ± 0.07c
Soluble Sugar (%)	3.25 ± 0.06b	5.61 ± 0.08a	3.03 ± 0.09c	2.82 ± 0.12d
Total phenol (mg GAE g^− 1^ DW)	3.79 ± 0.13c	3.91 ± 0.03c	5.37 ± 0.21b	5.99 ± 0.35a
Flavonoid (mg RE g^− 1^ DW)	0.56 ± 0.01b	0.60 ± 0.01a	0.51 ± 0.01c	0.59 ± 0.02a

Note: The data are presented as the means ± standard errors of three biological replicates. The different letters following the numbers indicate significant differences (*p* < 0.05) according to Duncan’s multiple range test

### Metabolomic analysis

**Fig. 1:**
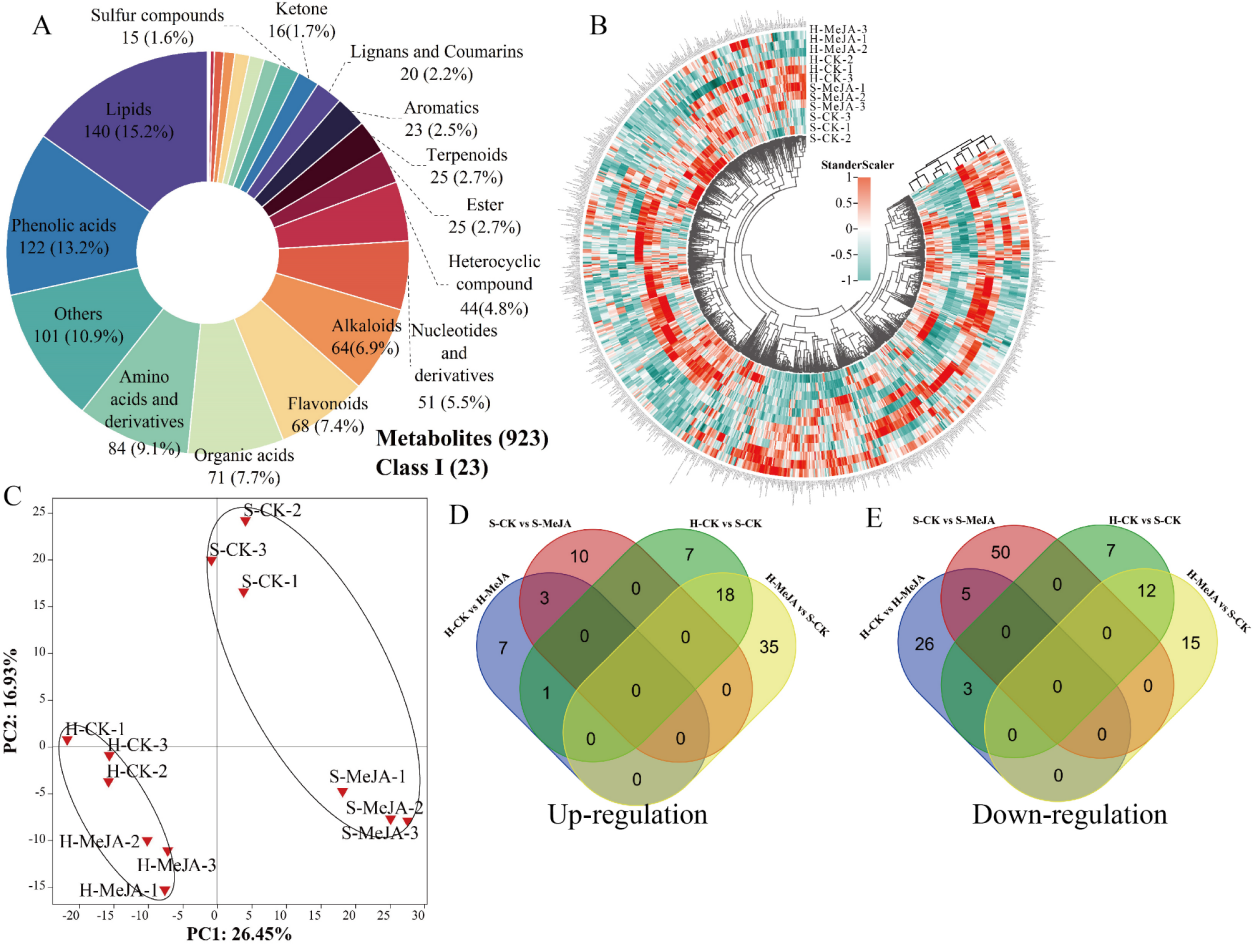
Metabolite overview (**A**), clustering heatmap (**B**), PCA (**C**), and Venn diagram of differentially abundant metabolites (**D** and **E**) for MeJA-treated Chinese chives analyzed via UPLC‒MS/MS and GC‒MS dual platforms. Different colors in panel A indicate primary classifications along with the number and percentage of detected metabolites. In the heatmap (**B**), each column represents a sample, each row represents a metabolite, red bars indicate high abundance, and green bars indicate low relative abundance. The volatile components were obtained from a previous study by Wang et al. (2023) [17]. In the circos plot, red represents high expression, and green indicates low expression

To examine metabolite accumulation changes in MeJA-treated substrates and hydroponic Chinese chives, 923 metabolites were detected via UPLC‒MS/MS and GC‒MS. These metabolites were classified into 23 categories (Fig. 1A). UPLC‒MS/MS identified 737 metabolites, including 19 terpenoids, 20 lignans, coumarins, 51 nucleotides and derivatives, 64 alkaloids, 68 flavonoids, 71 organic acids, 84 amino acids and derivatives, 98 others, 122 phenolic acids, and 140 lipids. GC‒MS identified 186 metabolites, including one ether, one acid, three halogenated hydrocarbons, three other compounds, six terpenoids, seven amines, eight phenols, and 11 alcohols. In addition, 11 aldehydes, 12 hydrocarbons, 15 sulfur-containing compounds, 16 ketones, 23 aromatic hydrocarbons, 25 esters, and 44 heterocyclic compounds were identified. A clustering heatmap was used to analyze the accumulation patterns of these 923 metabolites (Supplementary Table 1), revealing differences between MeJA-treated hydroponic and substrate-grown Chinese chives. The heatmap shows hierarchical clustering of the different cultivation methods and MeJA treatment groups (Fig. 1B). The H-CK, H-MeJA, S-CK, and S-MeJA treatments formed four categories, indicating variation in metabolite accumulation. Clustering of biological replicates demonstrated good homogeneity and reliability.

PCA was used to reveal the metabolomic features of the samples, enabling the observation of group differences in the PCA plots. The PCA score plot clearly revealed a separation between treatments, suggesting considerable changes in the metabolites of the MeJA-treated hydroponics and substrate-grown Chinese chives (Fig. 1, C). PC1 explained 26.45% of the variance in the 923 metabolite datasets, emphasizing differences in cultivation methods, whereas PC2 accounted for 16.93% of the variance related to MeJA treatment.

### Differentially abundant metabolite analysis

In the H-CK vs. H-MeJA groups, 45 differentially abundant metabolites were identified (11 upregulated and 34 downregulated); in the S-CK vs. S-MeJA groups, 68 (13 upregulated and 55 downregulated); in the H-CK vs. S-CK groups, 48 (26 upregulated and 22 downregulated); and in the H-MeJA vs. S-CK groups, 80 (27 upregulated and 53 downregulated) were identified (Fig. 1, D and E) (Supplementary Tables 2–5).

**Fig. 2:**
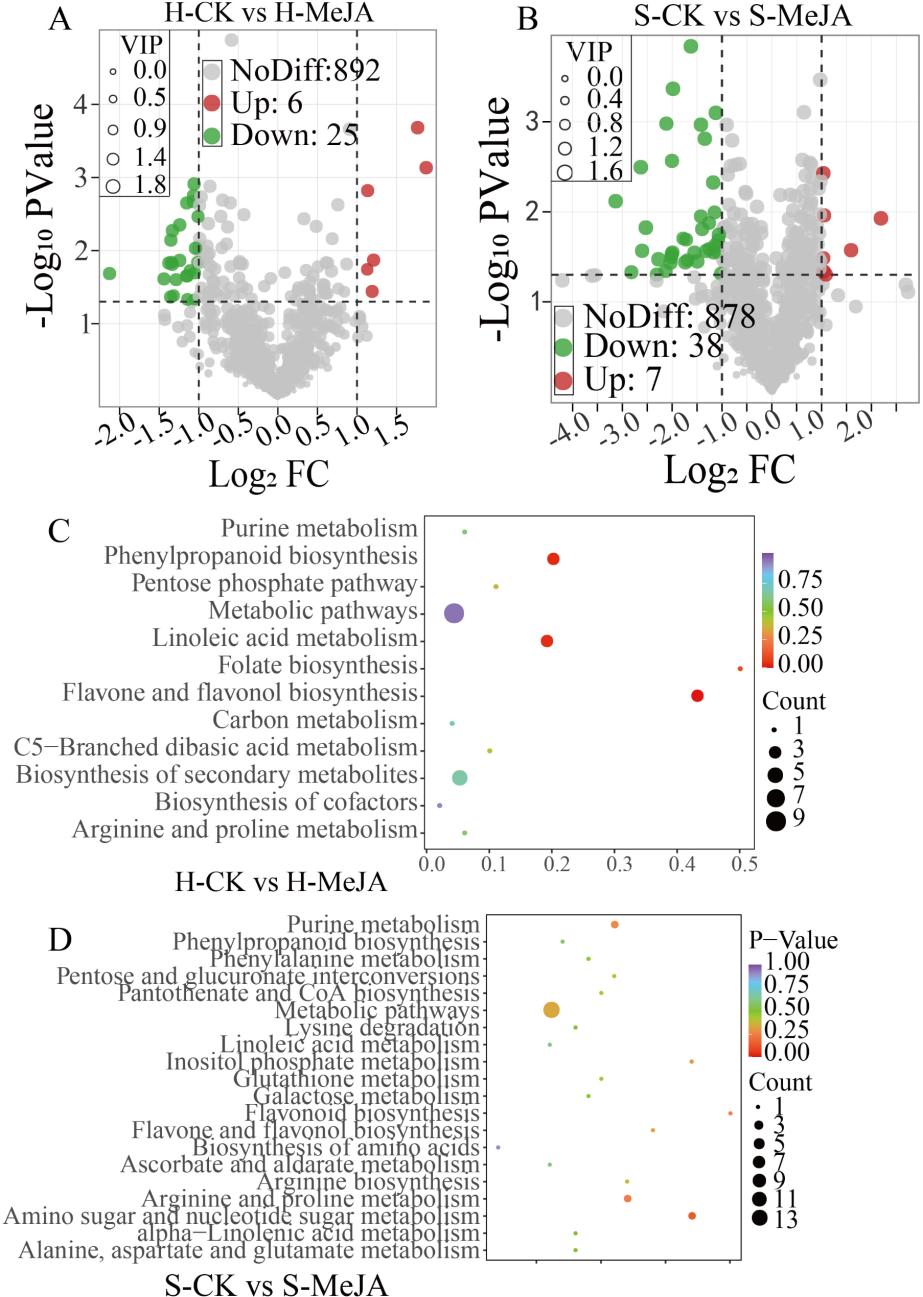
Differentially abundant metabolite analysis: The volcano plot features green dots for downregulated metabolites, red dots for upregulated metabolites, and gray dots for metabolites with nonsignificant differences. A larger absolute value on the x-axis indicates a greater difference in relative content between the two sample groups (**A** and **B**). For KEGG enrichment analysis of the differentially abundant metabolites (**C** and **D**), the x-axis represents the enrichment factor for each pathway, whereas the y-axis lists the pathway names. Dot color indicates the p value, with red indicating more significant enrichment, and the dot size reflects the number of enriched differentially abundant metabolites

Further refinement via Fold Change, VIP, and p value generated volcano plots (Fig. 2, A-B). In hydroponically cultivated Chinese chives, 31 differentially abundant metabolites were identified in the H-CK vs. H-MeJA comparison (6 upregulated, 25 downregulated), including significant upregulation of two phenolic acids, two lipids, one alkaloid, and one aldehyde and downregulation of one amino acid, two phenolic acids, one nucleotide, five flavonoids, one alkaloid, one organic acid, and 14 lipids (Fig. 2A). For substrate-cultured Chinese chives, 45 differential volatile compounds were identified in the S-CK vs. S-MeJA groups (7 upregulated, 38 downregulated), with significant upregulation of one terpene, one amino acid, three lipids, one ester, and one other compound. The downregulated genes included three amino acids, one amine, one alcohol, two aromatic hydrocarbons, two phenolic acids, five sulfur-containing compounds, one nucleotide, three flavonoids, five other classes, one aldehyde, two alkaloids, four organic acids, five heterocyclic compounds, and three esters (Fig. 2B).

Differentially abundant metabolites were cross-referenced with the KEGG database to determine their associated pathways. Enrichment analysis of the annotated results revealed pathways with significant enrichment of the differentially abundant metabolites. In the H-CK and H-MeJA groups, most metabolites were enriched in phenylpropanoid, linoleic acid, folate, and flavonoid biosynthesis (Fig. 2C). In the S-CK vs. S-MeJA groups, the metabolites were enriched in purine metabolism; phosphatidylinositol metabolism; flavonoid biosynthesis; arginine biosynthesis; arginine and proline metabolism; and amino and nucleotide sugar metabolism (Fig. 2D).

### Transcriptome sequencing and splicing of transcripts

To obtain transcriptional data for MeJA-treated hydroponic and substrate-grown Chinese chives, 12 RNA-Seq libraries from the H-CK, H-MeJA, S-CK, and S-MeJA samples were sequenced via the Illumina NovaSeq platform. Quality control via Fastp software revealed clean read percentages ranging from 96.4 to 97.7% (Supplementary Table 6). The mean Q20 and Q30 values were 97.79% and 93.61%, respectively, indicating high sequencing accuracy. The matching rate ranged from 76 to 80%, ensuring good transcriptome coverage.

The transcripts obtained through Trinity splicing served as references for further analysis (Supplementary Table 7). A total of 222,884 transcripts were generated, averaging 748 base pairs in length, with N50/N90 sizes of 1,080/325 and a total sequence base number of 166,807,729. Clustering with Corset produced 210,598 unigenes, averaging 748 bp in length, with N50/N90 sizes of 1106/343 and a total base count of 163,842,037.

### Functional annotation and classification of transcriptome assemblies in Chinese chives

Unigenes were annotated via the KEGG, Nr, Trembl, KOG, Gene Ontology (GO), SwissProt, and Pfam databases, with 34.74% (73,169) matching at least one sequence. A total of 210,598 unigenes were annotated (Supplementary Table 8). Compared with those in the NR library, the Chinese chive transcript sequences were highly similar to those in asparagus (33,307, 47.55%) and had fewer matches to those in oil palm (4,336, 6.19%), date palm (3,670, 5.24%), and other species (Supplementary Fig. 1A).

A total of 56,546 unigenes were assigned to at least one GO term (Supplementary Fig. 1B and Supplementary Table 9) and categorized into biological processes, cellular components, and molecular functions. The predominant biological processes included “cellular processes” (35,111), “metabolic processes” (31,376), and “stress responses” (14,416). The main cellular components were “cells” (40,925), “cell parts” (40,847), and “organelles” (31,621). The molecular function category included “binding” (33,439), “catalytic activity” (31,879), and “transporter protein activity” (4,428).

In the KOG functional classification, 42,600 unigenes were assigned to at least one category, with 4.5% being functionally unknown (Supplementary Fig. 1C). The major groups were “generic function prediction only” (10,322, 24.9%), “posttranslational modifications” (4,626, 10.9%), and “signal transduction mechanisms” (3,832, 9%). Other categories included “carbohydrate transport” (2,610, 6.1%), “translation” (2,382, 5.6%), “transcription” (2,254, 5.3%), and “RNA processing” (2,014, 4.7%).

### Differentially expressed gene screening and KEGG enrichment analysis

**Fig. 3:**
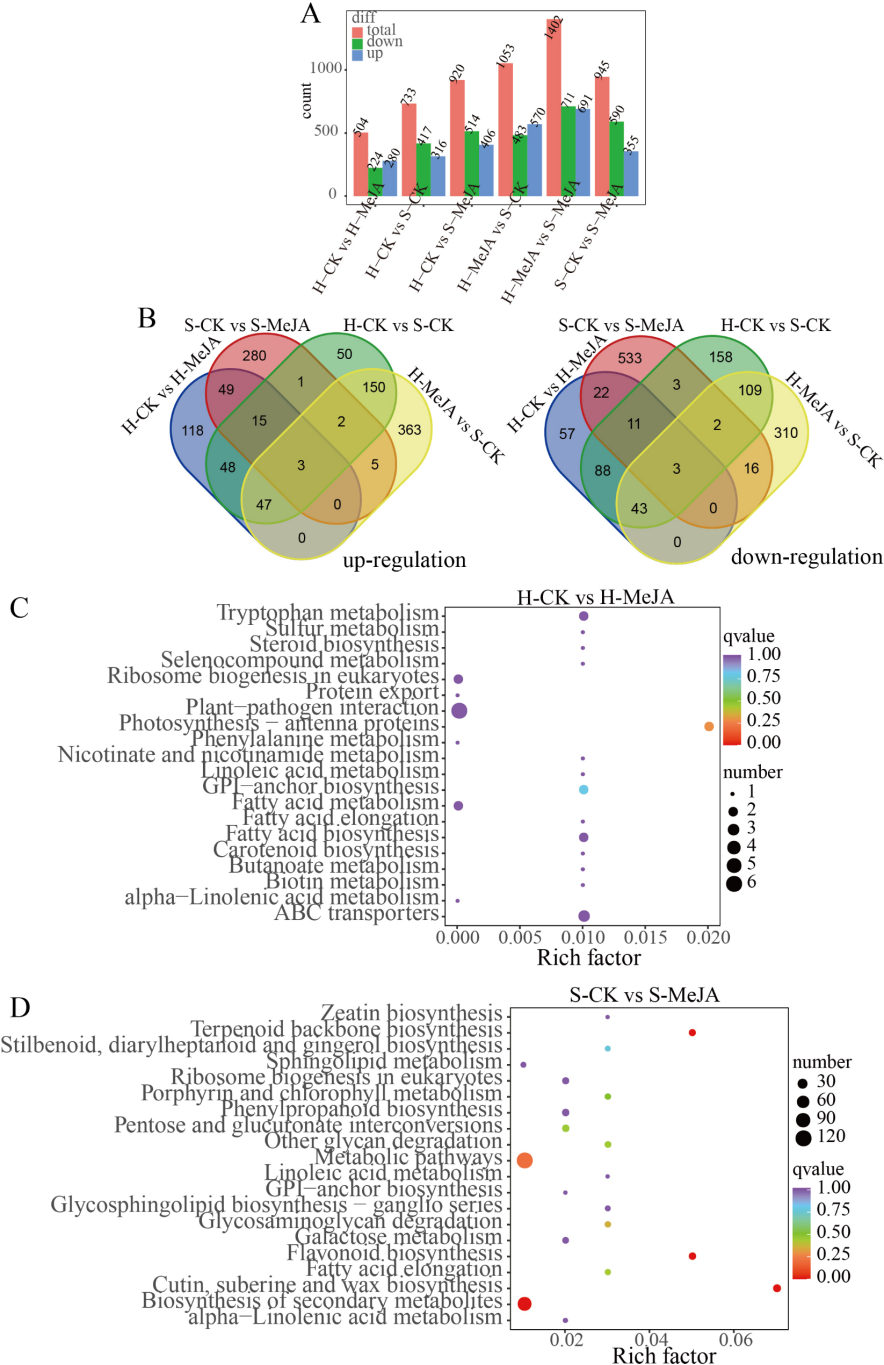
Differential gene expression screening and KEGG enrichment analysis: (**A**) The number of DEGs within each comparison group. (**B**) Upregulated and downregulated DEGs between H-CK and H-MeJA, S-CK and S-MeJA, H-CK and S-CK, and H-MeJA and S-CK. KEGG enrichment analysis of DEGs for H-CK vs. H-MeJA (**C**) and S-CK vs. S-MeJA (**D**). The y-axis indicates KEGG pathways, whereas the x-axis displays the rich factor; a higher rich factor signifies greater enrichment. Larger dots represent pathways with more DEGs, and a deeper red color indicates more significant enrichment

A total of 2,667 DEGs were identified. Specifically, 504 DEGs were identified (280 upregulated and 224 downregulated) in the H-CK vs. H-MeJA comparison. In the S-CK vs. S-MeJA comparison, 945 DEGs were identified (355 upregulated, 590 downregulated). In the H-CK vs. S-CK comparison, 733 DEGs were detected (316 upregulated, 417 downregulated). In the H-MeJA vs. S-CK comparison, 1,053 DEGs were observed (570 upregulated, 483 downregulated) (Fig. 3A and Supplementary Tables 11–14). In addition, 71 common DEGs were found between hydroponically grown H-CK and H-MeJA and between substrate-grown S-CK and S-MeJA, with 49 upregulated and 22 downregulated genes. Moreover, 259 common DEGs were identified in both the H-CK vs. S-CK and S-CK vs. S-MeJA comparisons, comprising 150 upregulated and 109 downregulated genes (Fig. 3B).

The Rich factor, q value, and number of differentially enriched genes quantified the degree of KEGG enrichment (Supplementary Tables 15–18). The top 20 most significantly enriched pathway entries were selected for display, or all if fewer than 20 were enriched (Fig. 3, C-D). KEGG enrichment analysis revealed plant signaling and biometabolic pathways involving the DEGs in MeJA-treated hydroponic and substrate-cultured Chinese chive leaves. The photosynthesis-antenna protein pathway was significantly enriched in the H-CK group compared with the H-MeJA group (q value < 0.05) (Fig. 3C). In the S-CK vs. S-MeJA comparison, terpene skeleton biosynthesis, metabolic pathways, flavonoid biosynthesis, the biosynthesis of secondary metabolites, and the biosynthesis of keratin, cork lipids, and waxes were significantly enriched (q value < 0.05), with metabolic pathways and the biosynthesis of secondary metabolites being the most enriched for the DEGs (Fig. 3D). These metabolic pathways provide insight into the metabolic processes of MeJA-treated hydroponic and substrate-grown Chinese chives.

### Integrating transcriptome and metabolome analysis

**Fig. 4:**
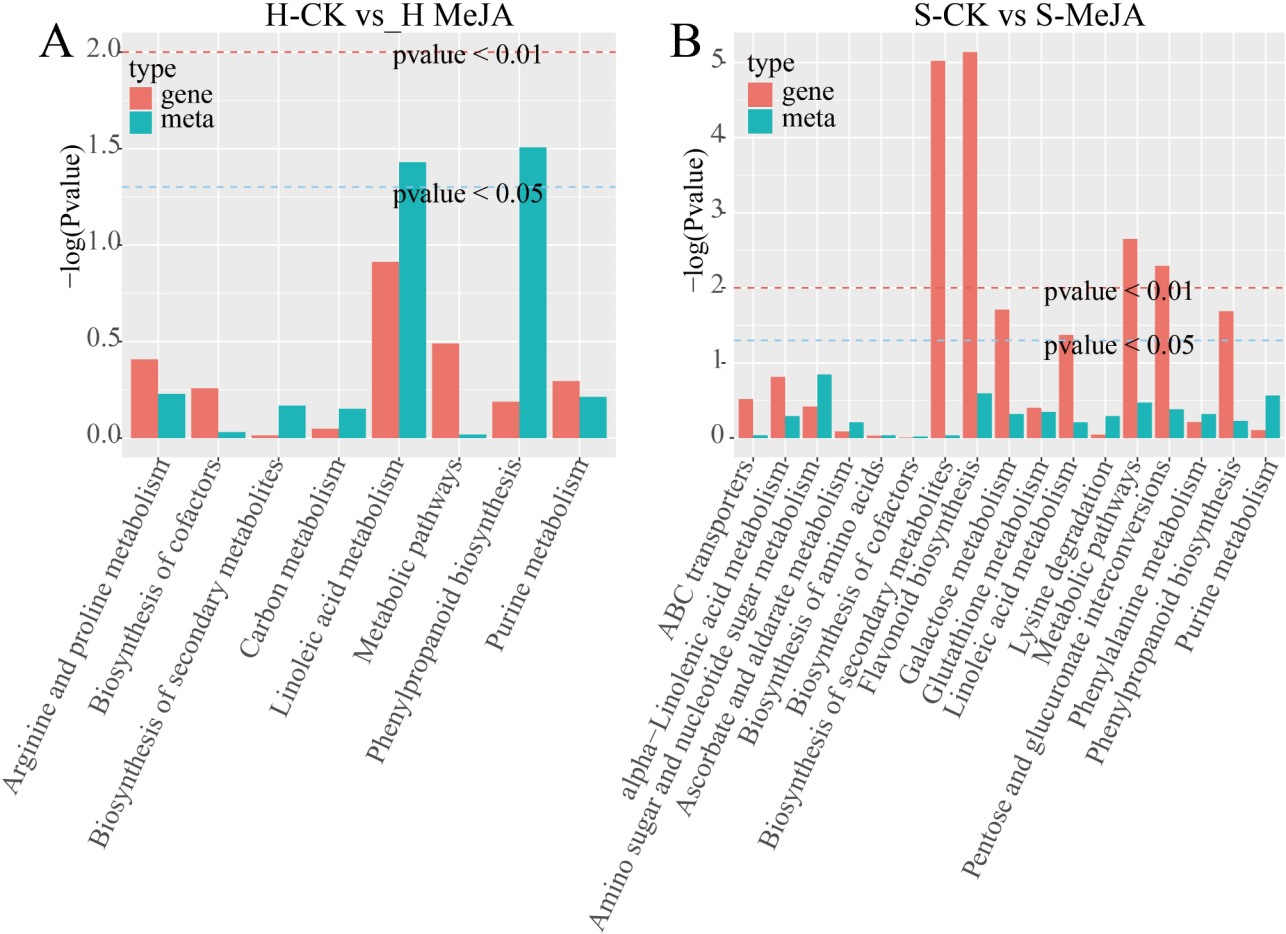
Integrating transcriptome and metabolome analysis for KEGG enrichment of differential metabolites and DEGs. The Abscissa of the bar chart represents the metabolic pathway, the red represents the enrichment pvalue of differential genes, and the green represents the enrichment p-value of differential metabolites, which is expressed by-log (*p*-value). The higher the ordinate, the stronger the enrichment

KEGG enrichment analysis of differentially abundant metabolites and DEGs (Supplementary Tables 19–22) revealed enriched pathways involving metastatic genes and differentially expressed genes. In the H-CK vs. H-MeJA comparison, the significant pathways included secondary metabolite biosynthesis (five DEGs, five metabolites), linoleic acid metabolism (one DEG, three metabolites), and phenylpropane biosynthesis (one DEG, three metabolites) (Fig. 4A). For the S-CK vs. S-MeJA comparison, the enriched pathways were pentose and glucuronide interconversion (11 DEGs, one metabolite), galactose metabolism (nine DEGs, one metabolite), linoleic acid metabolism (three DEGs, one metabolite), phenylalanine metabolism (12 DEGs, one metabolite), and metabolic pathways (123 DEGs, 14 metabolites) (Fig. 4B).

**Fig. 5:**
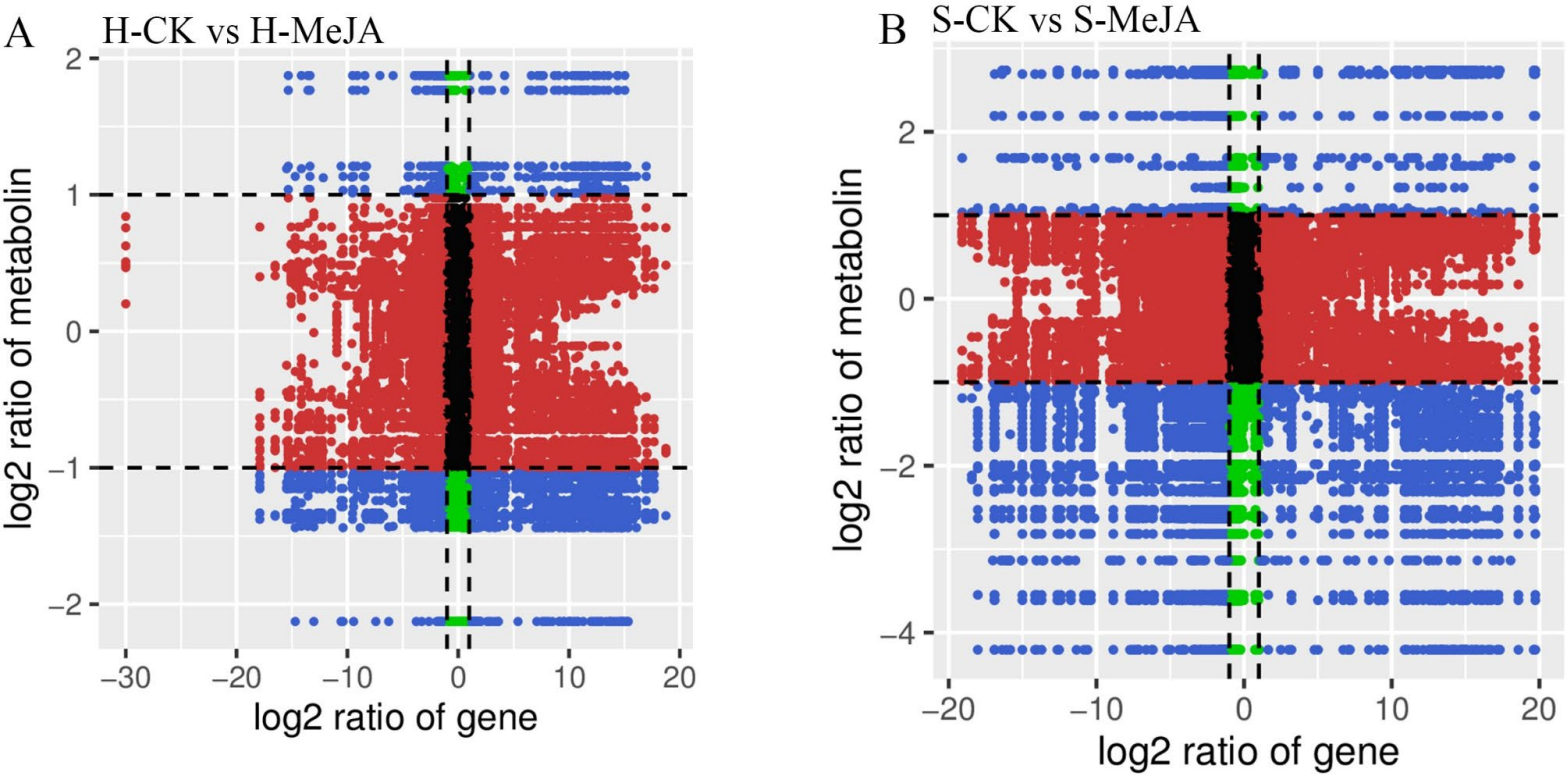
Pearson’s correlation analysis of genes and metabolites detected by the integrated transcriptome and metabolome for each differential grouping. From left to right, from top to bottom, it is divided into 1–9 quadrants. Limit 5: No differential expression of genes and metabolites; Limits 3 and 7: Consistent differential expression patterns of genes and metabolites; Limits 1 and 9: Differential expression patterns of genes and metabolites opposite; Limits 2, 4, 6 and 8: Metabolites are unchanged, genes are up-regulated or metabolites of unchanged genes are up-regulated

Correlation analysis of genes and metabolites in each group was conducted using R to calculate the Pearson correlation coefficient (PCC) [37]. A nine-quadrant plot was used to display fold changes of genes and metabolites with PCC > 0.8 in each differential group (Fig. 5). In quadrants three and seven, the DEG expression patterns matched the metabolite accumulation patterns, suggesting positive regulation by these genes. A total of 1,233 DEGs and 46 metabolites were identified in H-CK vs. H-MeJA (Fig. 5A), and 6,337 DEGs and 67 metabolites were identified in S-CK vs. S-MeJA (Fig. 5B). Furthermore, correlation analysis of DEGs and differential metabolites (PCC > 0.8) revealed that 691 DEGs and 43 metabolites were correlated in H-CK vs. H-MeJA, and 3,952 DEGs and 57 metabolites were correlated in S-CK vs. S-MeJA (Supplementary Fig. 2).

**Fig. 6:**
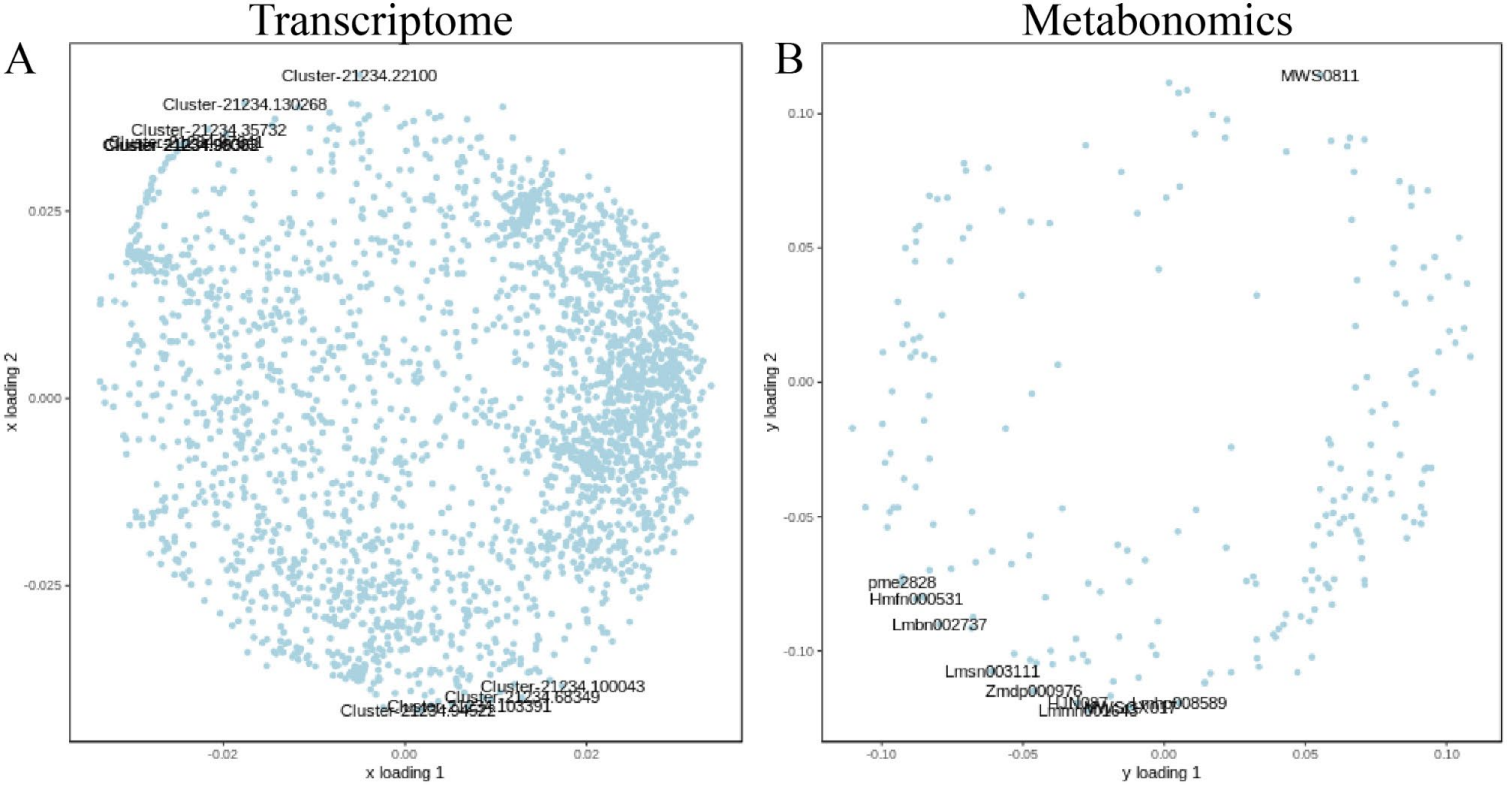
Loading diagram of O2PLS model established for all differential genes and differential metabolites. The distance from each point to the origin means the size of the correlation with another omics, and the top 10 genes (**A**) and metabolites (**B**) that have a greater impact on another omics are marked in the figure

**Fig. 7:**
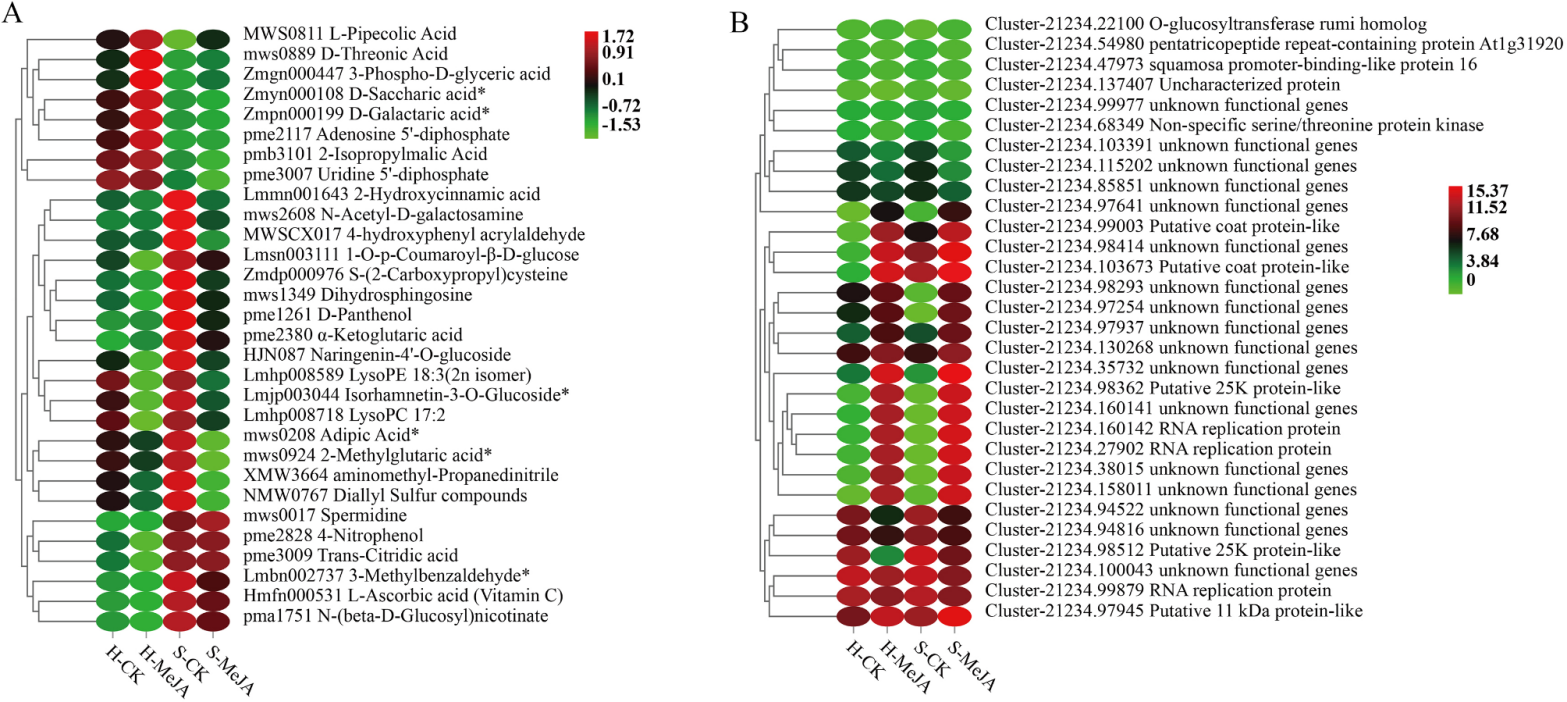
(**A**) Top 30 metabolites significantly affected by the transcriptome. (**B**) Top 30 genes significantly affected by metabolome. Red indicates high expression and green indicates low expression

Orthogonal Partial Least Squares (O2PLS), an unsupervised modeling approach, objectively describes potential associative trends between two datasets, minimizing the risk of false-positive associations at the source [37]. An O2PLS model was constructed using all DEGs and differential metabolites. Through the loading plot, variables with high correlation and weight across different datasets were preliminarily identified, enabling the screening of key variables influencing the other omics dataset (Fig. 6). The top 30 metabolites influenced by the transcriptome included various compounds (Fig. 7A). Among the top 30 genes affected by the metabolome, 15 presented high expression levels in MeJA-treated hydroponics and substrate-cultured Chinese chives compared with H-CK and S-CK, whereas five genes presented high expression levels in MeJA-treated Chinese chives (Fig. 7B).

### Exogenous MeJA induces the JA response pathway in Chinese chives

**Fig. 8:**
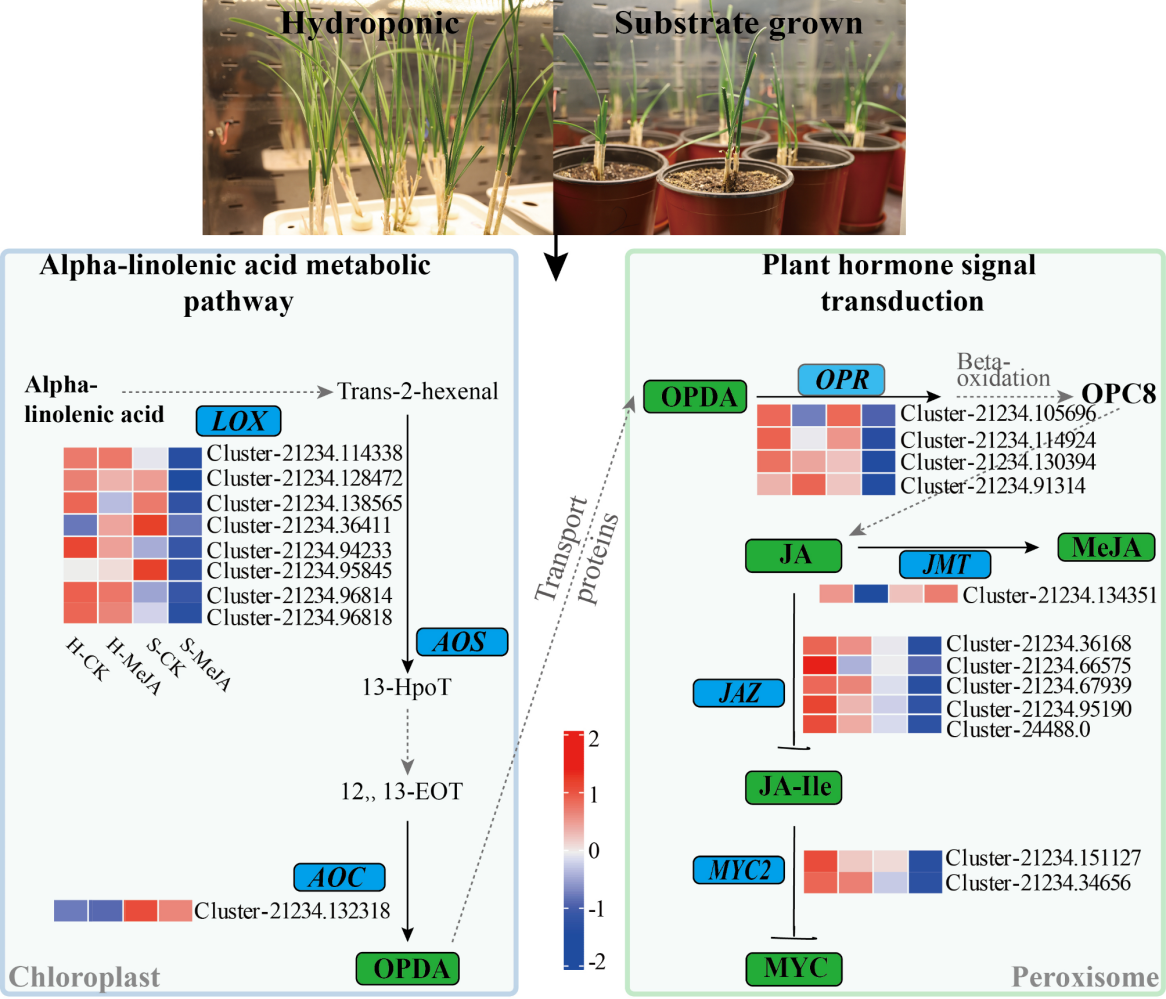
Heatmap of DEGs involved in the JA synthesis pathway. Blue represents high expression and red indicates low expression

Exogenous MeJA treatment caused the differential expression of 21 genes related to JA biosynthesis (Fig. 8). These genes included eight LOX genes (Cluster-21234.114338, Cluster-21234.128472, Cluster-21234.138565, Cluster-21234.36411, Cluster-21234.94233, Cluster-21234.95845, Cluster-21234.96814, Cluster-21234.96818), one AOC gene (Cluster-21234.132318), four OPR genes (Cluster-21234.105696, Cluster-21234.114924, Cluster-21234.130394, Cluster-21234.91314), one JMT gene (Cluster-21234.134351), five JAZ genes (Cluster-21234.36168, Cluster-21234.66575, Cluster-21234.67939, Cluster-21234.95190, Cluster-24488.0), and two MYC2 genes (Cluster-21234.151127, Cluster-21234.34656). Exogenous MeJA application inhibited JA biosynthesis and signaling in hydroponically grown Chinese chives, resulting in the downregulation of the LOX, AOC, OPR, JMT, JAZ, and MYC2 genes involved in the JA pathway.

### Analysis of the biosynthetic pathways of flavor substance precursors (CSOs)

**Fig. 9:**
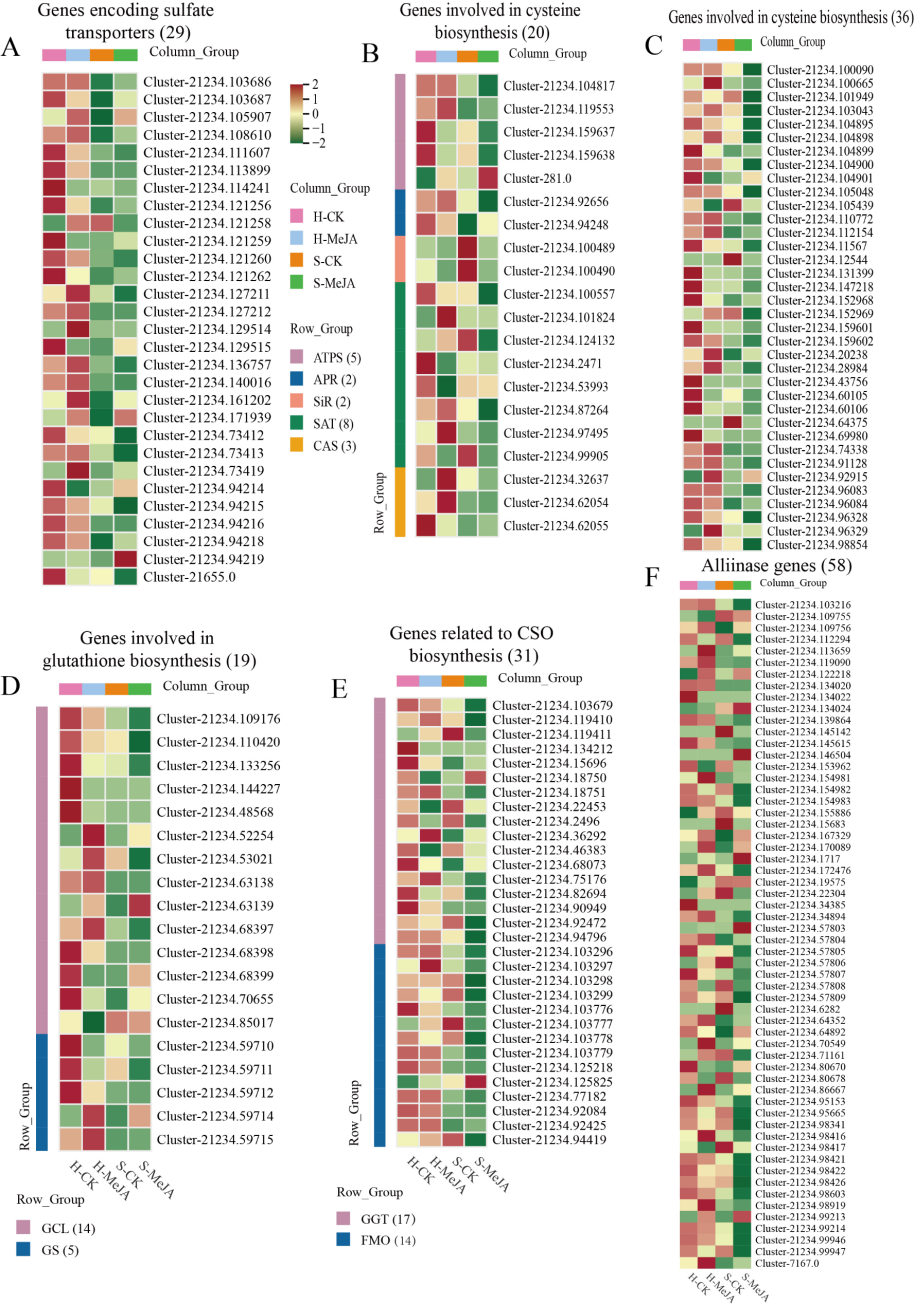
Expression analysis of genes related to the flavor formation pathway of Chinese chives. Red indicates high expression and green indicates low expression

**Fig. 10:**
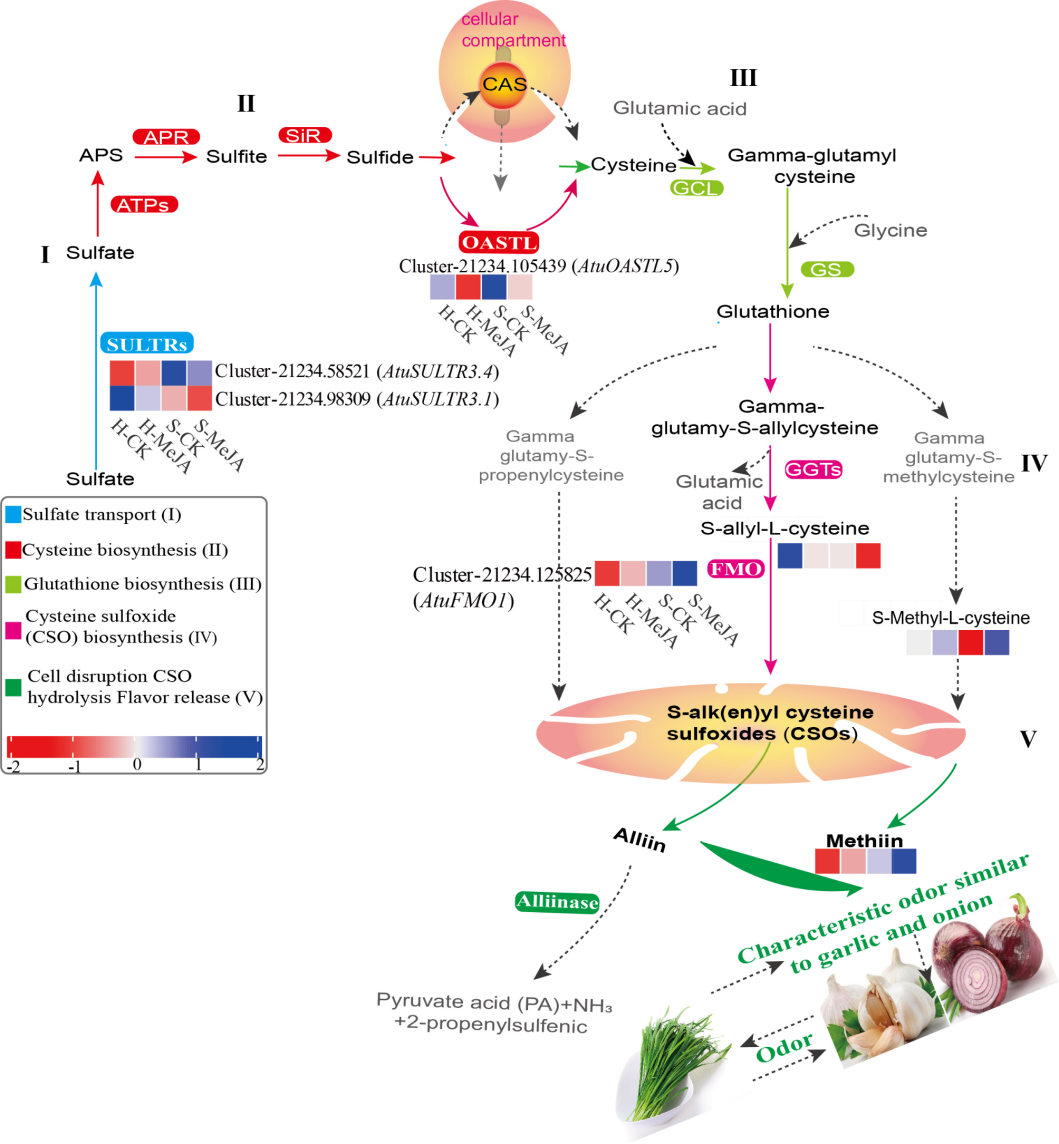
Analysis of DEGs and metabolite levels related to the biosynthesis of flavor precursor substances (CSOs) in MeJA-treated, soilless-cultured Chinese chives revealed key transporters and enzymes. Blue representing high expression and red indicating low expression.。

S-Alk(en)ylcysteine sulfoxides (CSOs), which are common in Allium plants, are key flavor precursors. To understand the role of MeJA treatment in flavor regulation in Chinese chives, we identified 193 CSO biosynthesis-related genes from the transcriptome data through a homology search and gene annotation (Fig. 9). Figure 10 shows the analysis of DEGs and metabolite levels in the CSO pathway in MeJA-treated soilless Chinese chives. Four DEGs associated with sulfate metabolism were identified: two SULTRs, one OASTL, and one FMO (Fig. 10). MeJA treatment upregulated *AtuSULTR1.1*/Cluster-21234.58521 in hydroponic Chinese chives and *AtuFMO1*/Cluster-21234.125825 in soilless Chinese chives. S-alkyl-L-cysteine levels in soilless Chinese chive leaves decreased significantly after MeJA application. The upregulated FMO gene (Cluster-21234.125825) increased the conversion of S-alkyl-L-cysteine to CSOs, increasing CSO accumulation (Fig. 10, IV-V). In addition, MeJA treatment elevated the levels of S-methyl-L-cysteine and methionine in hydroponically cultivated chive leaves.

### Analysis of FMO-regulated flavor substance precursor (CSO) biosynthesis

**Fig. 11:**
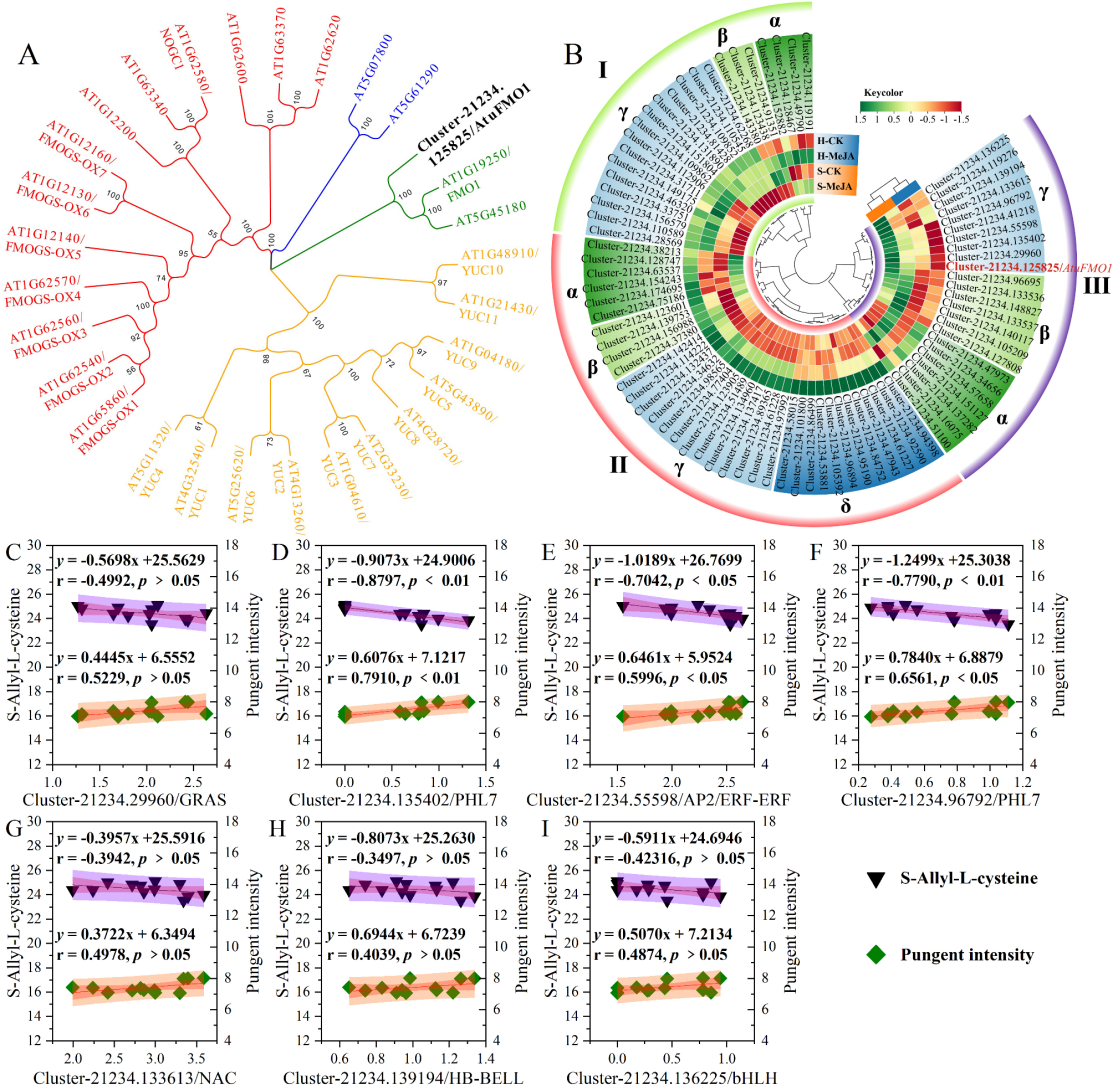
Analysis of the FMO enzyme-related differentially expressed gene Cluster-21234.125825 and its transcription factors (TFs) involved in the biosynthesis of flavor precursor CSOs in MeJA-treated, soilless-cultured Chinese chives included the following components: (**A**) A neighbor-joining tree was constructed to compare Cluster-21234.125825 with 29 FMO protein sequences from Arabidopsis. (**B**) Cluster analysis of Cluster-21234.125825 and differentially expressed TFs in treated chives. (**C**–**J**) Pearson correlation coefficients and linear regression analyses were conducted between the 12 transcription factors in II-α (Fig. 5B) and the synthesis precursor S-allyl-L-cysteine, as well as pungent flavor intensity, the latter of which was referenced from Wang et al. (2023) [17]. In the resulting circle heatmap, green signifies high expression, and red indicates low expression

FMO is an essential enzyme in CSO biosynthesis, making the inferred FMO gene Cluster-21234.125825 a candidate for detailed study. Phylogenetic analysis via the neighbor‒joining method with 28 FMO protein sequences from Arabidopsis and Cluster-21234.125825 revealed that Cluster-21234.125825 is closely related to At1G19250/*AtFMO1* (Fig. 11A). AtFMO1, a flavin-binding monooxygenase in *Arabidopsis*, suggested that *AtuFMO1*/Cluster-21234.125825 encodes a similar enzyme. Despite its low transcriptional abundance, *AtuFMO1* expression significantly increased with exogenous MeJA in hydroponically grown Chinese chives, indicating its crucial role in enhancing the flavor intensity of soilless-cultivated Chinese chives.

Eighty DEGs were identified as potential transcription factors (TFs) in the transcriptome analysis. The WRKY, AP2/ERF-ERF, MYB, and bHLH families responded to exogenous MeJA treatments in hydroponically grown Chinese chives. It has been hypothesized that *AtuFMO1* is targeted by multiple TFs with similar expression patterns. Cluster analysis revealed three TF groups whose expression differed in response to MeJA treatment (Fig. 11B). Clusters I and II were highly expressed in hydroponically grown Chinese chives but expressed at lower levels in substrate-cultivated Chinese chives. *AtuFMO1* and nine related TFs were categorized within the γ subbranch of Cluster III, indicating similar expression patterns (Fig. 11B). Pearson’s correlation analysis of the RNA-Seq data, S-alkyl-L-cysteine levels, and leaf pungency intensity revealed significant correlations (Fig. 11, C-I). Cluster-21234.135402/PHL7 was significantly negatively correlated with S-alkyl-L-cysteine accumulation (*r* = 0.8797, *p* < 0.01) (Fig. 11D), as were Cluster-21234.55598/AP2/ERF-ERF (*r* = 0.7042, *p* < 0.05) (Fig. 11E) and Cluster-21234.96792/PHL7 (*r* = 0.7790, *p* < 0.01) (Fig. 11F). Conversely, these TFs were positively correlated with leaf pungency intensity, particularly in Cluster-21234.135402/PHL7 (*r* = 0.7910, *p* < 0.01), Cluster-21234.55598/AP2/ERF-ERF (*r* = 0.5996, *p* < 0.05), and Cluster-21234.96792/PHL7 (*r* = 0.6561, *p* < 0.05). These results suggest that these TFs may promote the conversion of S-alkyl-L-cysteine to conjugated sulfur compounds (CSOs), increasing pungency. Thus, Cluster-21234.135402/PHL7, Cluster-21234.55598/AP2/ERF-ERF, and Cluster-21234.96792/PHL7 may act as positive regulators of *AtuFMO1* in MeJA-induced CSO biosynthesis.

### qRT‒PCR validation analysis

**Fig. 12:**
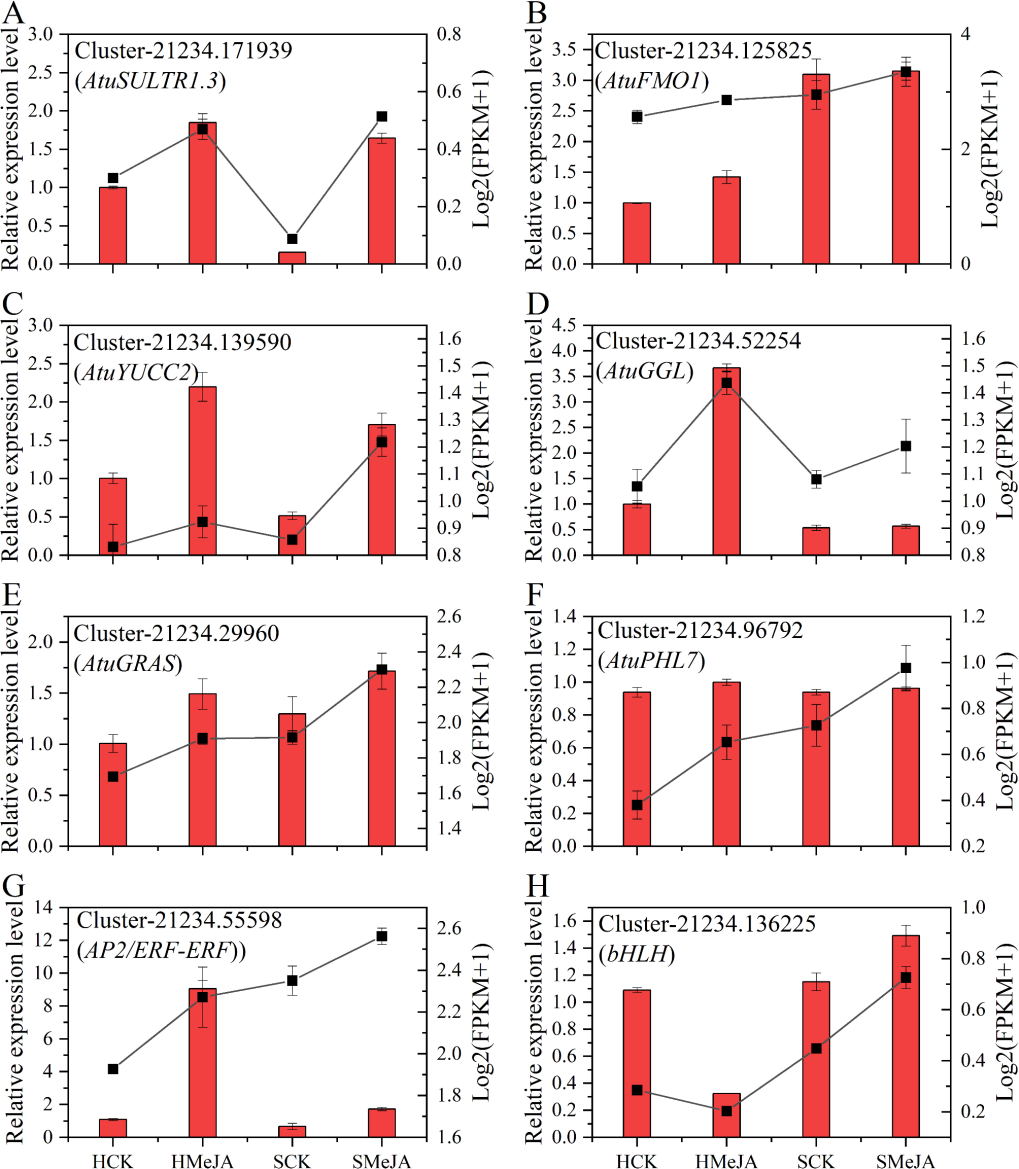
Quantitative qRT‒qPCR validations. (**A**–**H**) Relative expression of 8 genes. The bar graph shows the RT‒qPCR results, and the line graph shows the RNA‒Seq results. The scale on the left axis indicates the relative expression level, and the right axis indicates the Log2(FPKM + 1) value

Transcriptional profiles from RNA-Seq were validated by quantitative reverse transcription‒polymerase chain reaction (qRT‒PCR) in independent experiments. Eight genes were randomly selected for validation. Except for one gene (Cluster-21234.55598 (AP2/ERF-ERF)), the expression levels of the other seven genes matched the RNA-Seq data, showing 87.5% concordance (Fig. 12). Therefore, the qRT‒PCR results confirmed the reliability of the RNA-Seq-generated gene expression profiles.

## Discussion

Chinese chives are valued for their unique flavor profile and phytochemical composition [38], yet remain understudied compared to other Allium species. This study provides comprehensive analysis of metabolite profiles and regulatory mechanisms in hydroponic versus substrate-cultivated Chinese chives under MeJA treatment, identifying 923 metabolites classified into 23 groups (Fig. 1). Previous analyses of broadleaf and cultivated Chinese chives detected 341 metabolites through GC‒MS and LC‒MS [39], whereas LC‒MS of 30 garlic samples identified 472 metabolites [40]. Distinct hierarchical clustering patterns revealed significant variations in metabolite accumulation between different cultivation methods and MeJA treatment groups. In addition, these differences in flavor precursor compositions underscore the diversity among Allium species; specific precursor ratios are characteristic to each species [41]. The identification of S-allyl-l-cysteine and S-methyl-l-cysteine (Fig. 4) further aligns with established literature showing that allicin forms a predominant portion of the flavor profile in Allium species, where methionine plays a critical role [41]. Xia et al. (2022) reported that the methionine-to-allicin ratio in Chinese chives is approximately 6:1 [39]. Notably, this study’s detection of methionine and cyclic allicin but failed to detect allicin or isoallicin, despite their reported presence in Chinese chives [39]. This observation highlights the necessity of employing cultivation method-specific analytical protocols, along with advanced high-resolution mass spectrometry and precise analytical tools, for flavor compound analysis.

The KEGG enrichment analysis indicates that the production of bioactive compounds in hydroponically cultivated Chinese chives engages multiple metabolic pathways, including those involved in phenylpropanoid, flavonoid biosynthesis and linoleic acid metabolism (Fig. 2C). In contrast, substrate-cultivated Chinese chives utilize pathways centered on purine, flavonoid and arginine biosynthesis, and inositol phosphate metabolism (Fig. 2D). Glutathione, the main organic sulfur compound for transport and storage in plants, begins its biosynthesis of cysteine sulfoxide with the S-alkylation of glutathione. Importantly, glutathione metabolism, critical for sulfur compound biosynthesis, was enriched in both cultivation methods, suggesting that modifications in metabolic pathways due to MeJA treatments may enhance flavor formation through alterations in glutathione dynamics.

Allium plants are characterized by their richness in sulfur compounds, which contribute to both sensory properties and bioactivities, including antioxidant and antibacterial effects [42]. Transcriptomic analyses provided insight into the molecular mechanisms governing flavor development, identifying 193 candidate genes associated with cysteine sulfoxide (CSO) biosynthesis (Fig. 9). Hydroponic systems preferentially expressed Cluster-21234.127212 (*AtuSULTR1.2*), whereas substrate-grown Chinese chives upregulated Cluster-21234.94215 (*AtuSULTR1.2*) (Fig. 9). This isoform switching suggests adaptive regulation of sulfate uptake mechanisms, potentially optimizing sulfur acquisition under distinct root zone environments. Notably, the significantly higher SULTR expression observed in hydroponic Chinese chives indicates that MeJA application enhances sulfate transport capacity under controlled nutrient conditions, playing a crucial role in ensuring efficient production of flavor compounds. Downstream of sulfate uptake, the study revealed notable variations in key enzymatic steps within the sulfur assimilation pathway. During sulfate assimilation, transcripts such as Cluster-21234.119553 and Cluster-281.0 appear to regulate adenosine-5’-phosphosulfate production consistently across cultivation methods. However, in the cysteine biosynthesis pathway, distinct expression patterns of adenosine-5’-phosphosulfate reductase (APR) isoforms (e.g., Cluster-21234.92656 and Cluster-21234.94248, both annotated as *AtuAPR3*) underscore the influence of cultivation mode on metabolic fluxes. The impact of MeJA treatment is further evident in the regulation of genes involved in converting sulfite to organic sulfur compounds and, ultimately, in glutathione biosynthesis. The significant upregulation of *AtuSiR1* (Clusters 21234.100489 and 21234.100490) implies enhanced conversion efficiency of sulfite, while the elevated expression of *AtuGCL1* (Cluster-21234.110420) in soilless Chinese chives accentuates glutathione synthesis following elicitor application. Similarly, the GGT gene cluster (Cluster-21234.36292) and additional GGT genes (Clusters 21234.75176 and 21234.103679) showed marked induction in soilless Chinese chives, reinforcing their roles in CSO biosynthesis under MeJA treatment. These findings suggest that MeJA orchestrates sulfur flux toward flavor compound production. Furthermore, the hydrolysis of CSOs—catalyzed by alliinase enzymes that convert these non-volatile precursors into the volatile sulfur compounds responsible for Allium aroma—was significantly influenced by MeJA. Out of 58 alliinase genes identified, 13 were highly expressed in soilless Chinese chives post-treatment. This indicates that MeJA not only enhances sulfur metabolism but also modulates the final steps of flavor compound biosynthesis in soilless systems.

Moreover, 12-oxo-phytodienoic acid reductase (OPR) activity affects JA biosynthesis by modulating OPDA [43]. MeJA has also been shown to influence secondary metabolic pathways related to JA anabolism and signaling [44]. MeJA treatment significantly influenced JA-mediated signaling pathways, as evidenced by the enrichment of DEGs in “JA-mediated signaling pathway” (GO:0009867) and “cellular response to JA stimulus” (GO:0071395). This aligns with previous findings that exogenous MeJA reduces JA synthase activity (e.g., LOX, AOC, and OPR) and JA accumulation in Chinese chives [45]. Additionally, MeJA modulated secondary metabolic pathways, including flavonoid metabolism, sulfur ester metabolism, and lipid oxidation, as revealed by GO and KEGG enrichment analyses. These pathways are critical for metabolite accumulation and flavor compound biosynthesis, highlighting MeJA’s role as a regulator of primary and secondary metabolism. The observed inhibition of LOX, AOC, and OPR genes (Fig. 8) suggests a feedback suppression mechanism where exogenous MeJA downregulates endogenous JA biosynthesis. This paradoxical regulation contrasts with typical JA signaling patterns in model plants, possibly reflecting evolutionary adaptation in Allium species to prioritize secondary metabolite production over defense signaling when exposed to external elicitors that warrants further investigation. Crucially, MeJA simultaneously activated sulfur assimilation genes (SULTRs, OASTL) and *AtuFMO1* (Fig. 10), creating a metabolic channeling effect that directs sulfur flux toward CSO synthesis rather than general protein synthesis. Moreover, exogenous MeJA promoted growth, nutritional quality, antioxidant capacity and dry matter accumulation in Chinese chives (Table 1), demonstrating its potential application in improving agricultural practices.

The unique flavor of onion vegetables stems from the conversion of tasteless CSO precursors into volatile sulfur compounds by alliinase upon tissue disruption [46]. Although previous studies have characterized the CSO metabolic pathway in several Allium species [2, 47], the impact of external hormonal stimuli on CSO biosynthesis and the underlying regulatory mechanisms remain largely unexplored. This study provides critical insights into the molecular regulation of CSO biosynthesis in Chinese chives under exogenous MeJA treatment. Our results demonstrate that *AtuFMO1*, an enzyme analogous to *AsFMO1* in garlic [6], plays a pivotal role in mediating MeJA-induced CSO biosynthesis. Specifically, enhanced expression of *AtuFMO1*, in response to MeJA (Fig. 10), appears to drive the rate-limiting sulfur monooxygenation step required for S-allyl cysteine sulfoxide (ACSO) production. This finding reinforces the central role of FMO enzymes in linking hormonal signaling to flavor compound formation. Furthermore, our integrated transcriptomic and metabolomic analyses identified a significant coexpression of several transcription factor families—namely MYB, bHLH, NAC, and AP2/ERF—with *AtuFMO1* (Fig. 11B). In addition to *AtuFMO1*, MYB subfamily members, including MYB28, MYB34, and MYB51, play crucial roles in sulfur metabolism and abiotic stress induction [48]. bHLH TFs are essential for thioglucoside biosynthesis through the MYB-bHLH complex [49]. AP2/ERF transcription factors have been identified as potential regulators of MYB-bHLH complexes [50]. Notably, TFs such as Cluster-21234.135402 (PHL7), Cluster-21234.55598 (AP2/ERF-ERF), and Cluster-21234.96792 (PHL7) exhibited strong positive correlations with metabolites related to CSO biosynthesis. These regulatory factors may modulate sulfur metabolism by promoting the transcription of *AtuFMO1*, thereby enhancing the conversion of S-alkyl-L-cysteine into CSOs and intensifying the characteristic pungency of Chinese chives. Critically, while these correlations provide a compelling model for the transcriptional regulation of CSO biosynthesis under MeJA influence, the study has limitations. The functional roles of these candidate TFs, including AtuPHL7 and AtuAP2/ERF-ERF, remain to be verified at the protein level. Future investigations employing proteomic techniques, such as Western blotting or enzyme activity assays, are required to validate these interactions and elucidate the molecular mechanisms by which TFs regulate *AtuFMO*-mediated metabolism of garlic flavor compounds.

## Conclusions

Exogenous methyl jasmonate (MeJA) enhances the pungent flavor of soilless-cultivated Chinese chives by activating key enzyme-encoding genes (*AtuFMO1*) involved in the biosynthesis of CSOs, thereby increasing the accumulation of flavor precursors such as methiin and alliin. Our integrated metabolomic and transcriptomic analyses revealed that MeJA suppresses endogenous JA biosynthesis while promoting sulfur assimilation and CSO synthesis pathways. Notably, *AtuFMO1*, regulated by transcription factors AtuPHL7 and AP2/ERF-ERF, plays a pivotal role in catalyzing the sulfoxidation of S-alkyl-L-cysteine, a rate-limiting step in CSO production. These findings establish a framework for understanding how transcription factors (TFs) regulate *AtuFMO*-mediated metabolism of garlic flavor compounds, enhancing the pungent flavor of Chinese chives. This study highlights the potential of combining transcriptomic insights with agronomic practices to optimize flavor traits in Allium crops. Future research should focus on exploring the synergistic interactions between MeJA and other elicitors. Breeding strategies should focus on selecting Chinese chive varieties with high *AtuFMO1* expression and its associated TFs (e.g., AtuPHL7), utilizing marker-assisted selection or gene-editing tools to develop cultivars with inherently enhanced CSO biosynthetic capacity.

## Electronic supplementary material

Below is the link to the electronic supplementary material.

Supplementary Material 1

Supplementary Material 2

## Data Availability

Data are contained within the article or Supplementary Materials. The raw data of all RNA-Seq samples obtained in this study were deposited in the NCBI Sequence Read Archive under the project with identification number PRJNA1164635. The biological project and related SRA metadata can be obtained at the following website https://dataview.ncbi.nlm.nih.gov/object/PRJNA1164635?reviewer=k51rremqj2fosa9s6uq0a8qq4k.

## Abbreviations

CAD: Collision-activated dissociation
DEG: Differentially expressed gene
ESI: Electrospray ionization
GCL: Glutamate-cysteine ligase
GO: Gene Ontology
GS: Glutathione synthase
HCA: Hierarchical cluster analysis
JA: Jasmonic acid
MeJA: Methyl jasmonate
MS: Mass spectrometry
MSEA: Metabolite pooling enrichment analysis
OPDA: Oxophytodienoic acid
OPR: Oxo-phytodienoic acid reductase
PCA: Principal component analysis
PCC: Pearson correlation coefficient
SULTR: Sulfate transporter
TF: Transcription factor
UPLC: Ultra-performance liquid chromatography
