# Supplementary material for: Integrated metabolomics and transcriptomics analysis reveals the potential mechanism by which Methyl jasmonate enhances the pungent flavor of soilless-cultivated Chinese chives (Allium tuberosum)
Source: BMC Plant Biol. 2025 Mar 24;25:375. doi: 10.1186/s12870-025-06410-3 (PMC11931784; doi:10.1186/s12870-025-06410-3)
Supplement: Supplementary file 1 — Supplementary Material 1 [file 12870_2025_6410_MOESM1_ESM.pdf]

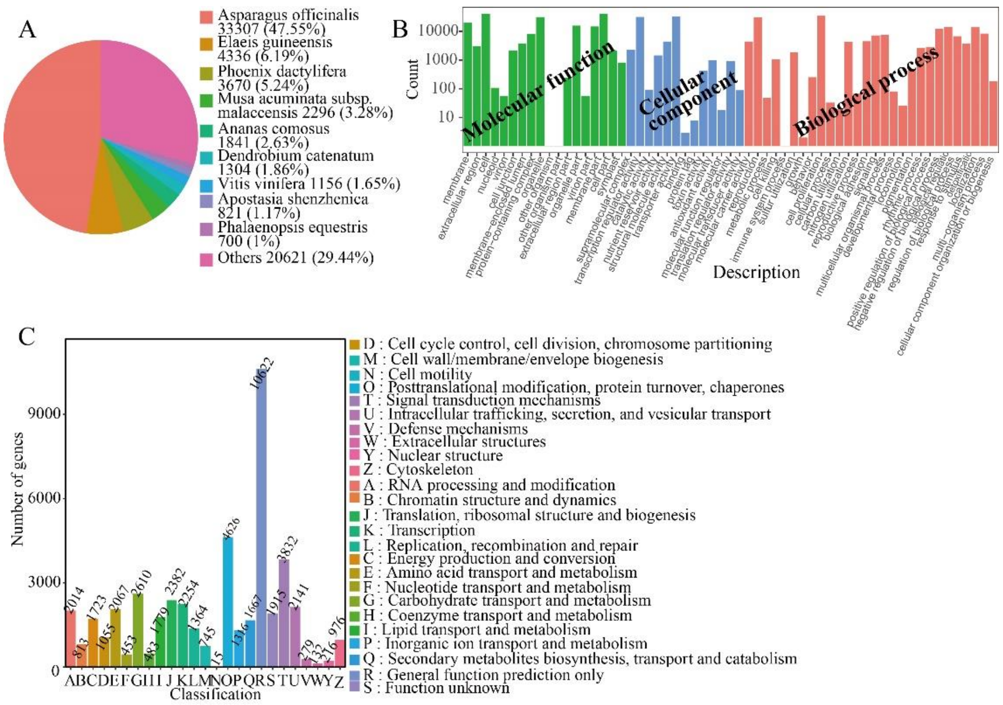

Supplementary Figure 1. Functional classification of the Chinese Chive transcriptome assembly. (A) NR database annotation results statistics. (B) Gene Ontology (GO) functional classification. The abscissa represents the secondary GO entry, and the ordinate represents the number of genes for the GO entry. (C) Orthologous relationship (KOG) functional distribution of the Chinese Chive transcriptome. The abscissa represents the classification content of KOG, and the ordinate represents the number of genes.

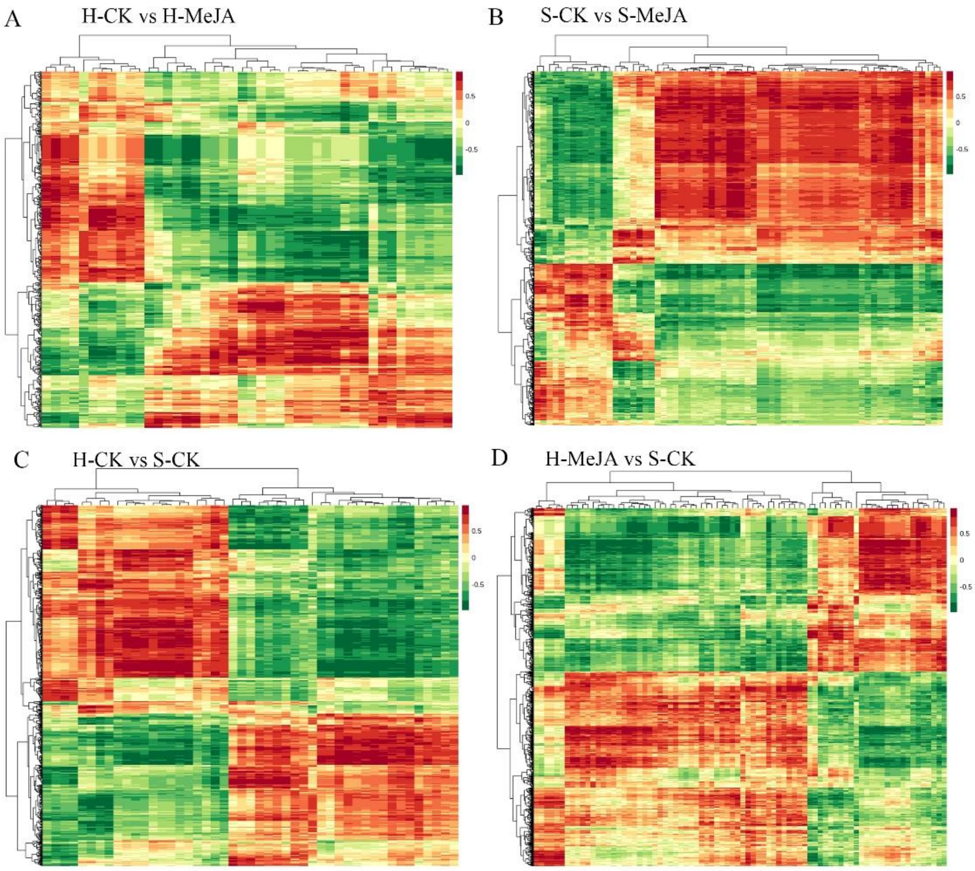

Supplementary Figure 2. Clustering heat map of the correlation coefficients of the integrated transcriptome and metabolome for differential genes and differential metabolites. The horizontal axis represents differential metabolites and hierarchical clustering results, and the vertical axis represents differential genes and hierarchical clustering results.
